# Effect of drug therapy on nerve repair of moderate-severe traumatic brain injury: A network meta-analysis

**DOI:** 10.3389/fphar.2022.1021653

**Published:** 2022-11-02

**Authors:** Mei Li, Xianhao Huo, Yangyang Wang, Wenchao Li, Lifei Xiao, Zhanfeng Jiang, Qian Han, Dongpo Su, Tong Chen, Hechun Xia

**Affiliations:** ^1^ Department of Neurosurgery, General Hospital of Ningxia Medical University, Yinchuan, China; ^2^ Ningxia Key Laboratory of Stem Cell and Regenerative Medicine, General Hospital of Ningxia Medical University, Yinchuan, China; ^3^ Department of Neurosurgery, North China University of Science and Technology Affiliated Hospital, Tangshan, Hebei, China; ^4^ School of Clinical Medicine, Ningxia Medical University, Yinchuan, China; ^5^ Ningxia Key Laboratory of Cerebrocranial Disease, Ningxia Medical University, Yinchuan, China

**Keywords:** drug therapy, favorable outcome indicators, mortality, network metaanalysis, traumatic brain injury

## Abstract

**Objective:** This network meta-analysis aimed to explore the effect of different drugs on mortality and neurological improvement in patients with traumatic brain injury (TBI), and to clarify which drug might be used as a more promising intervention for treating such patients by ranking.

**Methods:** We conducted a comprehensive search from PubMed, Medline, Embase, and Cochrane Library databases from the establishment of the database to 31 January 2022. Data were extracted from the included studies, and the quality was assessed using the Cochrane risk-of-bias tool. The primary outcome measure was mortality in patients with TBI. The secondary outcome measures were the proportion of favorable outcomes and the occurrence of drug treatment–related side effects in patients with TBI in each drug treatment group. Statistical analyses were performed using Stata v16.0 and RevMan v5.3.0.

**Results:** We included 30 randomized controlled trials that included 13 interventions (TXA, EPO, progesterone, progesterone + vitamin D, atorvastatin, beta-blocker therapy, Bradycor, Enoxaparin, Tracoprodi, dexanabinol, selenium, simvastatin, and placebo). The analysis revealed that these drugs significantly reduced mortality in patients with TBI and increased the proportion of patients with favorable outcomes after TBI compared with placebo. In terms of mortality after drug treatment, the order from the lowest to the highest was progesterone + vitamin D, beta-blocker therapy, EPO, simvastatin, Enoxaparin, Bradycor, Tracoprodi, selenium, atorvastatin, TXA, progesterone, dexanabinol, and placebo. In terms of the proportion of patients with favorable outcomes after drug treatment, the order from the highest to the lowest was as follows: Enoxaparin, progesterone + vitamin D, atorvastatin, simvastatin, Bradycor, EPO, beta-blocker therapy, progesterone, Tracoprodi, TXA, selenium, dexanabinol, and placebo. In addition, based on the classification of Glasgow Outcome Scale (GOS) scores after each drug treatment, this study also analyzed the three aspects of good recovery, moderate disability, and severe disability. It involved 10 interventions and revealed that compared with placebo treatment, a higher proportion of patients had a good recovery and moderate disability after treatment with progesterone + vitamin D, Bradycor, EPO, and progesterone. Meanwhile, the proportion of patients with a severe disability after treatment with progesterone + vitamin D and Bradycor was also low.

**Conclusion:** The analysis of this study revealed that in patients with TBI, TXA, EPO, progesterone, progesterone + vitamin D, atorvastatin, beta-blocker therapy, Bradycor, Enoxaparin, Tracoprodi, dexanabinol, selenium, and simvastatin all reduced mortality and increased the proportion of patients with favorable outcomes in such patients compared with placebo. Among these, the progesterone + vitamin D had not only a higher proportion of patients with good recovery and moderate disability but also a lower proportion of patients with severe disability and mortality. However, whether this intervention can be used for clinical promotion still needs further exploration.

## 1 Introduction

Traumatic brain injury (TBI) is defined as a change in brain function or other brain pathologies caused by external forces (Capizzi et al., 2020). The severity of this disease varies from mild to moderate and severe; among these, moderate and severe TBI are more likely to lead to mortality and poor functional outcomes than mild TBI (Jochems et al., 2021). It is a growing public health problem of major proportions. Statistics show that the incidence of TBI among the elderly people in high-income countries has increased more than expected, mainly due to falls (Brazinova et al., 2021). However, the increase in the use of motor vehicles in low- and middle-income countries has led to an increase in TBI caused by road traffic accidents, mainly affecting young people (Abdelmalik et al., 2019). TBI accounts for 30%–40% of all injury-related deaths in all age groups, indicating that neurological injury will remain the most crucial reason for disability caused by neurological diseases until 2030 (Maas et al., 2017).

The acquisition and reporting of TBI epidemiological data vary greatly worldwide, leading to significant differences in robust data reliability. The prediction of research data has revealed that more than 50 million new cases every year and more than 90% of these are mild TBIs (RennieRibecco-Lutkiewicz et al., 2018). However, in Europe, 2.5 million people suffer from TBI every year, 75,000 people die of the disease, and the incidence is about 200 per 100,000 people every year. In the United States, TBI leads to more than 280,000 hospitalizations, 2.2 million emergency department visits, and more than 52,000 deaths annually. Several large-scale, population-based studies conducted in the 1980s in China showed that the incidence of TBI was 55.4–64.1 cases per 100,000 people per year, equivalent to about 770,060–890,990 new cases per year (Ban et al., 2010; Gilchrist et al., 2011; Jiang et al., 2019; Khellaf et al., 2019). Although TBI treatment strategies have evolved significantly over the past 30 years, the mortality rate remains high. In addition, this disease costs the international economy about $400 billion annually, accounting for approximately 0.5% of the annual global output, given an evaluated standardized gross world product of $73.7 trillion (Humphreys et al., 2013). This undoubtedly brings a serious economic burden to their families, society, and countries. Therefore, TBI is a medical and public health problem that must be urgently addressed.

TBI can be pathologically divided into two stages: primary and secondary injuries. Primary injury refers to direct injury to the brain, while secondary injury may be caused by changes in cerebral blood flow and intracranial pressure, hypoxemia, systemic hypotension, and cerebral edema within a few hours after the injury (Robinson, 2021). The surgical removal of intracranial hematoma and ruptured brain tissue can reduce mortality, incidence of high intracranial pressure (ICP), and length of hospital stay in patients with TBI (Hyuk and Han, 2018; Lu et al., 2019).

However, surgery may also worsen the clinical outcome of TBI, partly due to the presence of secondary injury. Based on this, several drugs with neuroprotective effects have been studied for secondary injury after brain injury with varying degrees of success, mainly involving corticosteroids, progesterone, erythropoietin, amantadine, tranexamic acid, and cytarabine (Maas et al., 2006; Wright et al., 2007; Zafonte et al., 2012; Yutthakasemsunt et al., 2013; Nichol et al., 2015; Fattahi et al., 2018). However, the clinical trials exploring the neuroprotective effects of different drugs in patients with TBI involved comparisons with placebo; no direct clinical trials comparing different drugs have been conducted.

Network meta-analysis is an extension of traditional meta-analysis. It is based on the principle that the analysis can be achieved by setting up a common control group for indirect comparison of the effectiveness and safety of multiple interventions in similar diseases when direct comparative evidence is less or lacking (Jansen and Naci, 2013; Catala-Lopez et al., 2014). Therefore, this research group attempted to use the indirect comparison principle of network meta-analysis. Data on all the direct or indirect comparative treatment interventions of neuroprotective drugs used to reduce mortality were collected to improve the prognosis and neurological function of patients with TBI. The summary analysis was conducted to explore the role of different drug treatment schemes in TBI, and to determine which drugs might be used as a more promising intervention for treating such patients by ranking.

## 2 Patients and methods

### 2.1 Inclusion and exclusion criteria

The inclusion and exclusion criteria of this study were based on the PICOS strategy (P: Patient/population; I: Intervention; C: Comparison/control; O: Outcome; S: Study design). We included patients aged at least 15 years who suffered from TBI with a Glasgow coma score of 3–12 and the injury within 24 h. The treatment group used drugs such as corticosteroids, progesterone, erythropoietin, amantadine, tranexamic acid, citicoline, and so on; the control group received a placebo. The analysis included only randomized controlled clinical trials with a sample size of at least 10 people. The outcome indicators were mortality, and neurological function recovery rate (which were based on the GOS score).

The exclusion criteria were as follows: trials containing nontraumatic brain injury from other causes (e.g., cerebral hemorrhage, cerebral infarction, etc.), pregnant women, lactation, patients with a history of severe drug and food allergies, patients with severe heart, liver, or renal dysfunction, chronic alcohol or drug abuse, and severe psychiatric disorders; single case reports; single-arm trials without controls; and related trials without outcome indicators.

### 2.2 Result measurement

The primary outcome indicator was mortality in patients with TBI in each drug treatment group and control group. The secondary outcome indicators were GOS score (divided into five scales: 1 dead, 2 vegetative state, 3 severe disabilities, 4 moderate disability, and 5 Good recovery) used to evaluate the neurological function recovery rate of patients with TBI in different groups after each drug treatment, proportion of patients with favorable result (favorable result = good recovery + moderate disability), and adverse effects associated with different drug treatments.

### 2.3 Search strategy

The retrieval formula {[(traumatic brain injury) OR (traumatic head injury)] AND [(corticosteroids OR progesterone OR erythropoietin OR amantadine OR (tranexamic acid) OR citicoline OR (medical treatment) OR (drug treatment)] AND [(randomized controlled trial) OR random*)]} was used to conduct a comprehensive search in the databases of PubMed, Cochrane Library, Embase, and Medline from the establishment of the database to 31 January 2022. Meanwhile, relevant references, published systematic reviews, articles included in the meta-analysis, abstracts of conference papers, and ongoing or completed unpublished trials in the World Health Organization clinical registries were searched manually.

### 2.4 Data screening and quality evaluation

The inclusion and exclusion criteria were applied by two reviewers. They independently screened the literature-retrieval results and used the Cochrane quality evaluation method to assess all randomized trials from six aspects: randomization, allocation concealment, blind, selective bias, incomplete data, selective reporting, and other biases. In the case of randomization, the study was classified as having a “low” risk of bias if randomization was carried out with an appropriate method. The study could not be classified as having a high or low risk of bias, and if insufficient information regarding the implementation process was available, the study was defined instead as having an “unclear” risk of bias. The study was categorized as having a “high” risk of bias if randomization was not performed. Any problems or disagreements encountered during the screening of studies for inclusion in the analysis and quality assessment were resolved by two reviewers after consultation or by a third person through consultation.

### 2.5 Data extraction

Data were extracted from all included studies as follows: author, publication year, country, study type, age, intervention measures, number of people in each intervention group, details of drug treatment, mortality rates in patients with TBI in different treatment groups, rates of neurological recovery at the end of treatment, favorable outcome indicators, and incidence of side effects associated with each drug treatment. If data were missing, we contacted the corresponding author of the study wherever possible.

### 2.6 Statistical analysis

The heterogeneity test was carried out for all the included studies. When *p* > 0.1 and *I* ^2^ < 50%, the results were considered non-heterogeneous, and the fixed-effects model was adopted. Otherwise, the random-effects model was adopted. We used the two-tailed statistical test and considered the difference statistically significant when *p* < 0.05. In the network meta-analysis, we used the surface under the cumulative ranking curve (SUCRA) to rank the treatment effects. In addition, before combining direct and indirect evidence, the node-splitting method was used to conduct a consistency test to determine whether the two could be combined. The statistical analyses were performed using RevMan 5.3 (Cochrane Collaboration, London, United Kingdom) and Stata 16.0 (StataCorp, TX, United States).

## 3 Results

### 3.1 Literature search results

**FIGURE 1:**
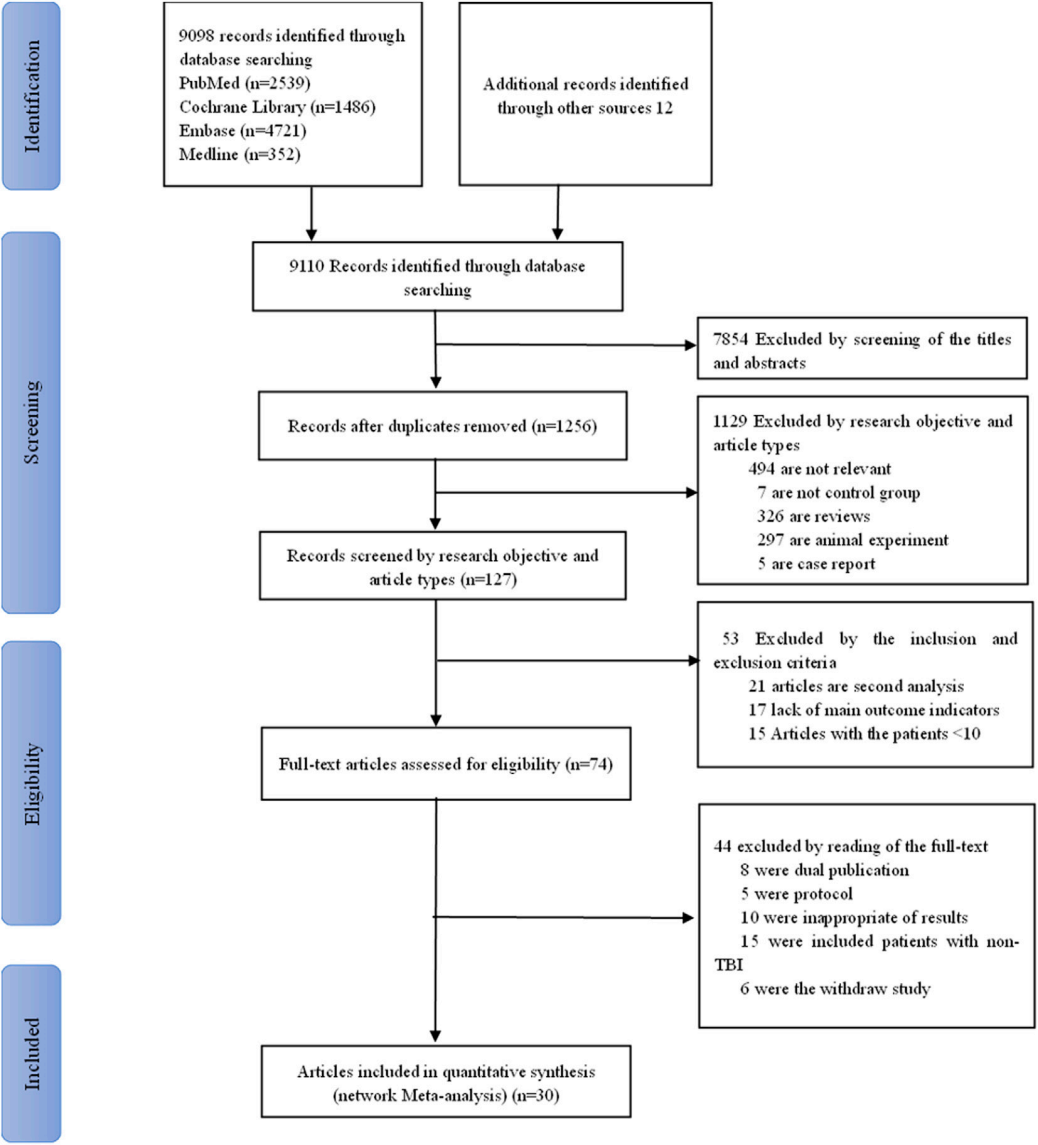
Flow chart of the study selection process.

A total of 9,110 studies were retrieved. First, 7,854 duplicate studies were removed by reading titles and abstracts. Then, 1,256 studies were screened by reading the research objective and article type. Consequently, 1,129 studies were excluded (because they were irrelevant, did not involve a control group, or were reviews, animal studies, or case reports). Based on the inclusion and exclusion criteria, we screened 127 studies and excluded 53 of them (because they were second analyses, lacked main outcome indicators, or involved a sample size of at least 10). Finally, after excluding 44 (studies due to dual publication, or protocols, or inappropriate results, and so on), the remaining 30 studies were included for network meta-analysis. The screening flow chart is shown in Figure 1. The data including study design, publication years, types of interventions, and other details are shown in Tables 1, 2. The total sample size was 26,956, and the study included 13 interventions, in the order of TXA (Yutthakasemsunt et al., 2013; Chakroun-Walha et al., 2018; Collaborators, 2019; collaborators et al., 2010; Fakharian et al., 2018; Mojallal et al., 2020; Rowell et al., 2020; Perel et al., 2012), EPO (Aloizos et al., 2015; Nichol et al., 2015; Li et al., 2016; Hellewell et al., 2017; Bai and Gao, 2018; Skrifvars et al., 2018), progesterone (Wright et al., 2007; Xiao et al., 2007; Xiao et al., 2008; Shakeri et al., 2013; Skolnick et al., 2014; Wright et al., 2014; Soltani et al., 2017), progesterone + vitamin D (Aminmansour et al., 2012), atorvastatin (Farzanegan et al., 2017), beta-blocker therapy (Khalili et al., 2020), Bradycor (Marmarou et al., 1999), Enoxaparin (Baharvahdat et al., 2019), Tracoprodi (Yurkewicz et al., 2005), dexanabinol (Maas et al., 2006), selenium (Moghaddam et al., 2017), simvastatin (Shafiee et al., 2021) and placebo.

**TABLE 1:** Baseline characteristics of involved patients.

Study	Country	Publication year	Age(y)	Male (I/C%)	GCS	Cases	Outcomes	Follow-up time
Yutthakasemsunt S	Thailand	2013	>16	86%/91%	4–12	120/120	Mortality, functional status by using GOS, thromboembolic events	NR
Marmaractou A	North America	1999	16–70	71%/82%	3–8	66/67	Mortality, functional status by using GOS, adverse effect	6 months
Yurkewicz L	United States	2005	16–70	73.7%/73.7%	4–8	198/206	Mortality, functional status by using GOS, adverse effect	6 months
Maas AI	Multinational	2006	16–65	80%/83%	3–12	428/418	Mortality, functional status by using GOS, adverse effect	6 months
Wright DW	United States	2007	>16	71%/70%	4–12	77/23	Mortality, functional status by using GOS, adverse effect	1 month
Perel P	United Kingdom	2012	>16	83.5%/85.4%	3–12	133/137	Mortality, functional status by using GOS	
Shakeri M	Iran	2013	18–65	NR	3–8	38/38	Mortality, functional status by using GOS	3 months
Li ZM	China	2016	≥16	49%/41%	3–8	75/71	Mortality, functional status by using GOS, adverse effect	3 months
Moghaddam OM	Iran	2017	≥18	78.9%/80.4%	4–12	57/56	Mortality, functional status by using GOS	2 months
Soltani Z	Iran	2017	18–60	NR	3–12	20/24	Mortality, functional status by using GOS	6 months
Fakharian E	Iran	2017	≥16	90.5%/88%	3–12	74/75	Mortality, functional status by using GOS	3 months
Chakroun-Walha O	Tunisia	2019	≥18	89.2%/91.6%	3–12	96/84	Mortality, functional status by using GOS, adverse effect	1 month
CRASH-2 trial collaborators	Multinational	2010	NR	NR	3–12	3,138/3,174	Mortality	1 month
CRASH-3 trial collaborators	Multinational	2019	≥16	80%/80%	3–12	4,649/4,553	Mortality, adverse	NR
Mojallal F	Iran	2020	≥18	71.4%/90.9%	3–12	56/44	Mortality	NR
Rowell SE	United States and Canada	2020	≥15	73.5%/75%	3–12	657/309	Mortality, functional status by using GOS, adverse effect	6 months
Xiao GM	China	2007	15–65	Total 58.9%	5–8	26/30	Mortality	3 months
Xiao GM	China	2008	18–65	70%/74%	3–8	82/77	Mortality, functional status by using GOS, adverse effect	6 months
Aminmansour B	Iran	2012	NR	80%/80%/60%	≤8	20/20/20	Mortality, functional status by using GOS	3 months
Skolnick BE	Multinational	2014	16–70	78.5%/78.7%	3–8	591/588	Mortality, functional status by using GOS, adverse effect	6 months
Wright DW	United States	2014	≥17	73.3%/74.1%	4–12	442/440	Mortality, functional status by using GOS, adverse effect	6 months
Nichol A	multinational	2015	≥16	84%/83%	3–12	305/298	Mortality, functional status by using GOS, adverse effect	6 months
Aloizos S	Greece	2015	18–65	95.8%/88.9%	3–9	24/18	Mortality	6 months
Skrifvars MB	multinational	2018	≥18	NR	3–12	305/297	Mortality, functional status by using GOS	6 months
Bai XF	China	2018	18–70	68.3%/73.3%	<8	60/60	Mortality, functional status by using GOS, adverse effect	10 weeks
Hellewell SC	Australia	2018	15–65	70%/67%	3–12	23/21	Mortality, functional status by using GOS	6 months
Farzanegan GR	Iran	2017	18–75	95.2%/87.0%	5–13	21/23	Mortality, functional status by using GOS	3 months
Shafiee S	Iran	2021	18–60	65.3%/69.4%	≤8	49/49	Mortality, functional status by using GOS, adverse	NR
Baharvahdat H	United States	2019	16–70	84.6%/81.5%	5–8	26/27	Mortality, functional status by using GOS	NR
Khalili H	United States	2020	≥18	86.9%/85.8%	≤12	99/120	Mortality, functional status by using GOS	6 months

GCS, glasgow coma score; GOS, glasgow outcome score; I, intervention; C, control; NR, not report.

**TABLE 2:** Overview of the included literature.

Study	Publication year	Study design	Study type	Intervention control	Initiation of intervention	Length of intervention	Treatment details
Yutthakaseactmsunt S	2013	Randomized double-blind, placeco-controlled parallel group trial	Monocenter	TXA placebo	Intervention within 8 h of injury	NR	TXA (loading dose of 1.0 g over 30 min, followed by a maintenance dose of 1.0 g infused over 8 h) or placebo
Marmarou A	1999	Phase II prospective randomized, double-blind, clinical trial	Multicenter	Bradycor Placebo	Intervention within 12 h of injury	5 days	Intravenous infusion of Bradycor 3 ug/kg/min or placebo
Yurkewicz L	2005	Randomized, double-blind, placebo controlled trial	Multicenter	Tracoprodil placebo	Intervention within 8 h of injury	3 days	Intravenous infusion of traxoprodil for 2 h at 0.75 mg/kg/h then continuing for 70 h at 0.37 mg/kg/h, or placebo
Maas AI	2006	Phase III randomized, double-blind, placeo controlled trial	Multicenter	Dexanabinol placebo	Intervention within 6 h of injury	NR	Intravenous injection of 150 mg dexanabinol or placebo
Wright DW	2007	Phase II randomized double-blind placebo-controlled trial	Monocenter	Progesterone placebo	Intervention within 12 h of injury	3 days	Loading dose of 0.71 mg/kg of progesterone at 14 ml/h for the first hour. Then, the infusion was reduced to 10 ml/h to deliver 0.5 mg/kg per hour for the next 11 h
Perel P	2012	Double-blind randomized placebo-controlled trial	Multicenter	TXA placebo	Intervention within 8 h of injury	NR	Loading dose of 1 g of TXA infused over 10 min followed by an infusion of 1 g over 8 h or matching placebo
Shakeri M	2013	Single blinded randomized control experiment	Monocenter	Progesterone placebo	Intervention within 8 h of injury	5 days	Progesterone or placebo was taken every 12 h, 1 mg/kg gavaged *via* nasogastric tube
Li ZM	2016	Randomized double-blind controlled trial	Monocenter	EPO placebo	Intervention within 8 h of injury	12 days	Subcutaneous injection of 100 units/kg EPO or placebo
Moghaddam OM	2017	Randomized double-blind, placebo, controlled trial	Monocenter	Selenium placebo	Intervention within 8 h of injury	14 days	Selenium 500 ug.or placebo as a continuous Intravenous infusion
Soltani Z	2017	Randomized single-blind placebo, controlled trial	Monocenter	Progesterone placebo	Intervention within 4 h of injury	5 days	Progesterone or placebo was given intramuscularly 1 mg/kg every 12 h
Fakharian E	2017	Randomized double-blind, placebo, controlled trial	Monocenter	TXA placebo	Intervention within 8 h of injury	NR	Intravenous TXA with the first dose of 1 g in 100 ml of normal saline and then with a maintenance dose of 1 g per 1000 ml of normal saline for 8 h or placebo
Chakroun-Walha O	2019	Randomized open-label trial	Monocenter	TXA placebo	Intervention within 24 h of injury	NR	Intravenous TXA with the first dose of 1 g in 100 ml of normal saline in 10 min and then with a maintenance dose of 1 g per 500 ml of normal saline or placebo
CRASH-2 trial collaborators	2010	Randomized placebo-controlled trial	Multicenter	TXA placebo	Intervention within 8 h of injury	NR	1 g of tranexamic acid by intravenous infusion or placebo
CRASH-3 trial collaborators	2019	Randomized placebo-controlled trial	Multicenter	TXA placebo	Intervention within 3 h of injury	NR	1 g of tranexamic acid *via* intravenous infusion or placebo
Mojallal F	2020	Randomized double-blind controlled trial	Monocenter	TXA placebo	Intervention within 8 h of injury	NR	Intravenous TXA with the first dose of 1 g in 100 ml of normal saline in 10 min and then with a maintenance dose of 1 g per 500 ml of normal saline or placebo
Rowell SE	2020	Randomized double-blind, phase Ⅱ trial	Multicenter	TXA placebo	Intervention within 2 h of injury	NR	1 g IV tranexamic acid and then 1 g tranexamic acid IV infusion over 8 h/2 g IV tranexamic acid, or IV placebo
Xiao GM	2007	Randomized controlled trial	Monocenter	Progesterone placebo	Intervention within 24 h of injury	5 days	Progesterone or placebo 1.0 mg/lg *via* intramuscular injection, twice a day
Xiao GM	2008	prospective, randomized, placebo-controlled, double-blind	Monocenter	Progesterone placebo	Intervention within 8 h of injury	5 days	Progesterone or placebo 1.0 mg/kg *via* intramuscular injection, twice a day
Aminmansour B	2012	Randomized, placebo-controlled trial	Monocenter	Progesterone + vitamin D progesterone placebo	Intervention within 8 h of injury	5 days	1 mg/kg of progesterone intramuscularly every 12h, and the second group also 5ug/kg vitamin D once-a-day, or placebo
Skolnick BE	2014	Prospective, randomized, double-blind, parallel-group trial	Multicenter	Progesterone placebo	Intervention within 8 h of injury	5 days	Intravenously with Progesterone or placebo, 0.71 mg/kg per hour for 1 h, followed by 0.5 mg/kg per hour for 119 h
Wright DW	2014	Randomized, double-blind, placebo-controlled clinical trial	Multicenter	Progesterone placebo	Intervention within 4 h of injury	4 days	Progesterone or placebo infused continuously with 14.3 ml/h for 1 h and then with 10 ml/h for 71 h; the dose was then tapered by 2.5 ml/h every 8 h
Nichol A	2015	Randomized double-blind, parallel-group, placebo-controlled trial	Multicenter	EPO placebo	Intervention within 24 h of injury	NR	Subcutaneous injection epoetin alfa 40000 IU or placebo
Aloizos S	2015	Randomized, double-blind crial	Multicenter	EPO placebo	Intervention within 8 h of injury	7 days	Erythropoietin (10,000 IU) or placebo
Skrifvars MB	2018	Randomized double-blind, parallel-group, placebo trial	Multicenter	EPO placebo	Intervention within 24 h of ICU admission	NR	Weekly doses of 40,000 IU of subcutaneous EPO or placebo
Bai XF	2018	Randomized controlled trial	Monocenter	EPO placebo	Intervention within 2 h of admission	15 days	Received erythropoietin 6000 IU or placebo by a subcutaneous injection
Hellewell SC	2018	Single-center randomized controlled trial	Multicenter	EPO placebo	Intervention within 24 of injury	15 days	EPO 40,000 IU or placebo by a subcutaneous injection
Farzanegan GR	2017	Randomized controlled trial	Monocenter	Atorvastatin placebo	Intervention within 10 h of injury	10 days	20 mg/d atorvastatin or placebo
Shafiee S	2021	Randomized double-blinded placebo-controlled trial	Monocenter	Simvastatin placebo	NR	10 days	Oral 40 mg/d simvastatin or placebo
Baharvahdat H	2019	Randomized double-blinded placebo-controlled trial	Monocenter	Enoxaparin placebo	Intervention within 5 h of injury	NR	Enoxaparin 0.5 mg/kg by subcutaneous injection or placebo
Khalili H	2020	Randomized non-blinded trial	Monocenter	Beta-blocker placebo	Intervention within 24 h of injury	10 days	Oral 20 mg of beta-blocker therapy or placebo, twice a day

TXA, tranexamic acid; EPO, erythropoietin; NR, not report.

### 3.2 Quality evaluation

**FIGURE 2:**
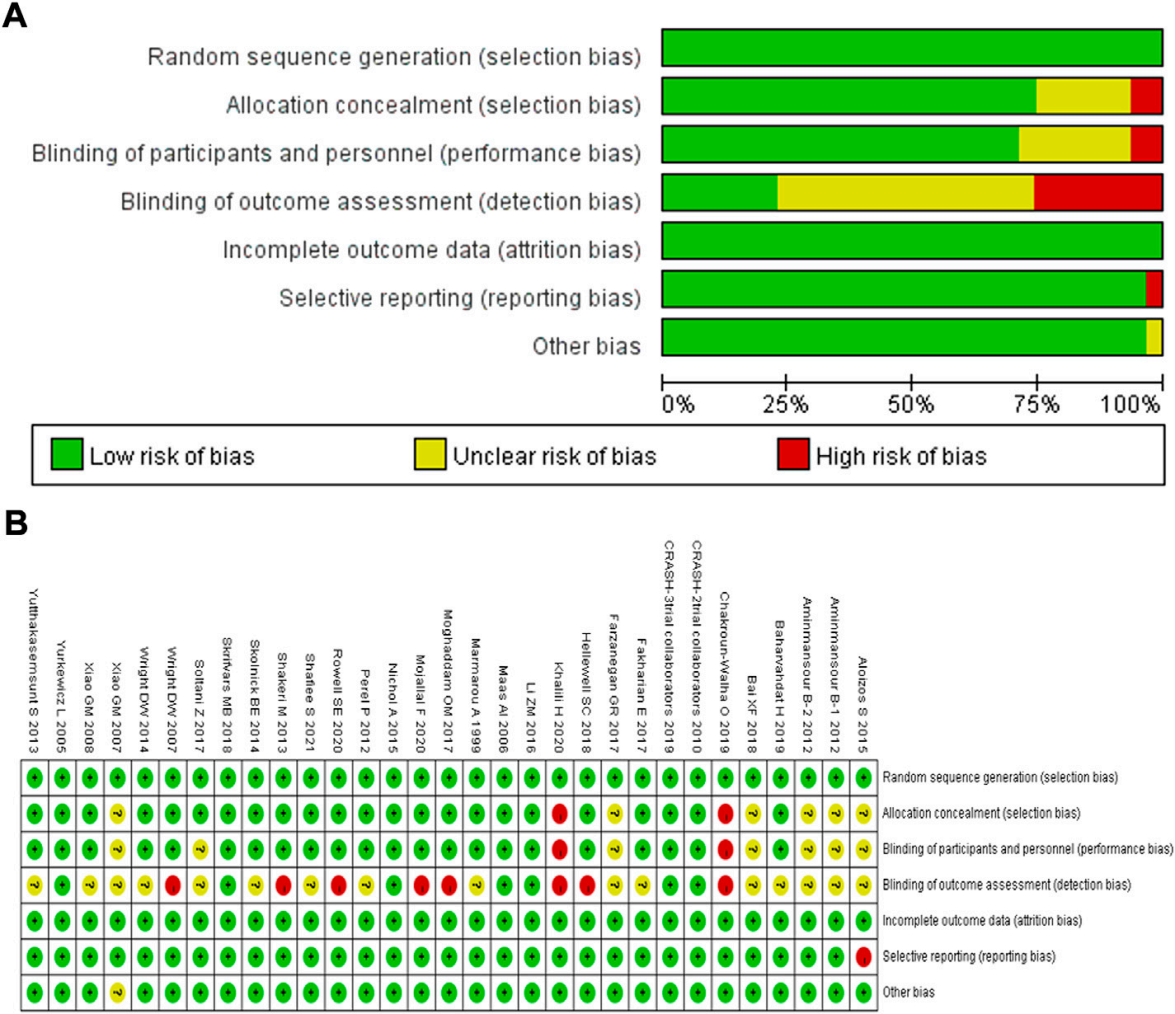
Quality assessment of identified randomized controlled trials; **(A)** Each risk of bias item presented as percentages across all included studies; **(B)** Each risk of bias item for each included study. Green indicates a low risk of bias, yellow indicates an unclear risk of bias, and red indicates a high risk of bias.

The 30 randomized controlled trials were included in the analysis. All the studies adopted the correct randomization method, and the data of the results were complete. Only Aloizos et al. (2015) had selective reports. For Aloizos et al. (2015), Aminmansour et al. (2012), Farzanegan et al. (2017), Bai and Gao, (2018), and Xiao et al. (2007), the correctness of the implementation of allocation concealment and blinding was uncertain. For Baharvahdat et al. (2019), Fakharian et al. (2018), Marmarou et al. (1999), Perel et al. (2012), Shafiee et al. (2021), (Skolnick et al. (2014), Wright et al. (2014), Xiao et al. (2008)), and Yutthakasemsunt et al. (2013), whether the correct blinding was applied in terms of outcome assessment was uncertain. Hellewell et al. (2017), Moghaddam et al. (2017), Mojallal et al. (2020), Wright et al. (2007), Rowell et al. (2020), and Shakeri et al. (2013) did not implement blinding for outcome assessment, and Chakroun-Walha et al. (2018) and Khalili et al. (2020) did not perform allocation concealment and implement blinding. This indicated that the quality of the randomized controlled studies included in the analysis was moderate (Figures 2A,B).

### 3.3 Traditional meta-analysis

The subgroup analysis in terms of mortality in patients with TBI after drug treatment revealed (Supplementary Figure S1) no heterogeneity between subgroups (*I* ^2^ < 50%, *p* > 0.1), and the fixed-effects model was adopted. The analysis showed that TXA and EPO treatment schemes significantly reduced mortality in patients with TBI compared with placebo treatment, with statistically significant differences (*p* = 0.009 and *p* = 0.003). However, compared with placebo treatment, progesterone, progesterone + vitamin D, Bradycor, Tracoprodil, dexanabinol, selenium, atorvastatin, simvastatin, Enoxaparin, and beta-blocker therapy did not reduce mortality in patients with TBI, and the differences were not statistically significant (all *p* > 0.05).

The subgroup analysis in terms of the proportion of patients with TBI having favorable outcomes after drug treatment revealed (Supplementary Figure S2) significant heterogeneity among the subgroups (*I* ^2^ > 50%, *p* < 0.1), and the random-effect models was adopted. The study revealed that Enoxaparin and Progesterone + vitamin D treatment schemes significantly improved the prognosis of patients with TBI compared with placebo treatment, with statistically significant differences (*p* = 0.03 and *p* = 0.04). However, compared with placebo treatment, Bradycor, progesterone, selenium, TXA, EPO, dexanabinol, Tracoprodi, atorvastatin, simvastatin, and beta-blocker -therapy did not significantly improve the prognosis of patients with TBI, and the differences were not statistically significant (all *p* > 0.05).

**FIGURE 3:**
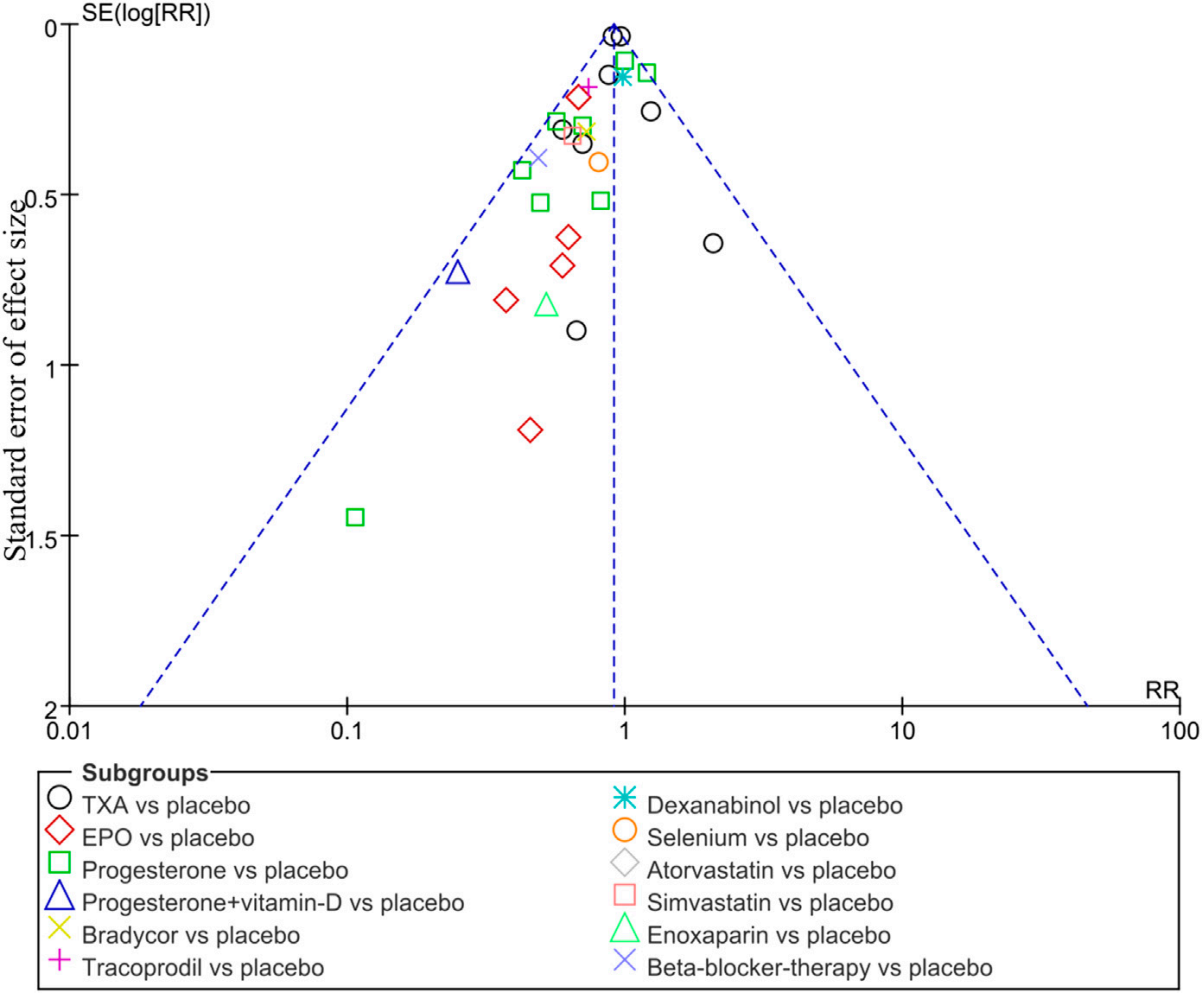
Funnel plots for the detection of publication bias on the mortality of TBI.

A funnel plot analysis was performed on the mortality after the two interventions for assessing publication bias, which was drawn with the RR value of each outcome as the horizontal coordinates and SE (log[RR]) as the longitudinal coordinates. The funnel plot was basically symmetrical, indicating no evidence of publication bias for the comparison and the results were statistically robust (Figure 3).

### 3.4 Network meta-analysis

#### 3.4.1 Network diagram of different intervention measures

**FIGURE 4:**
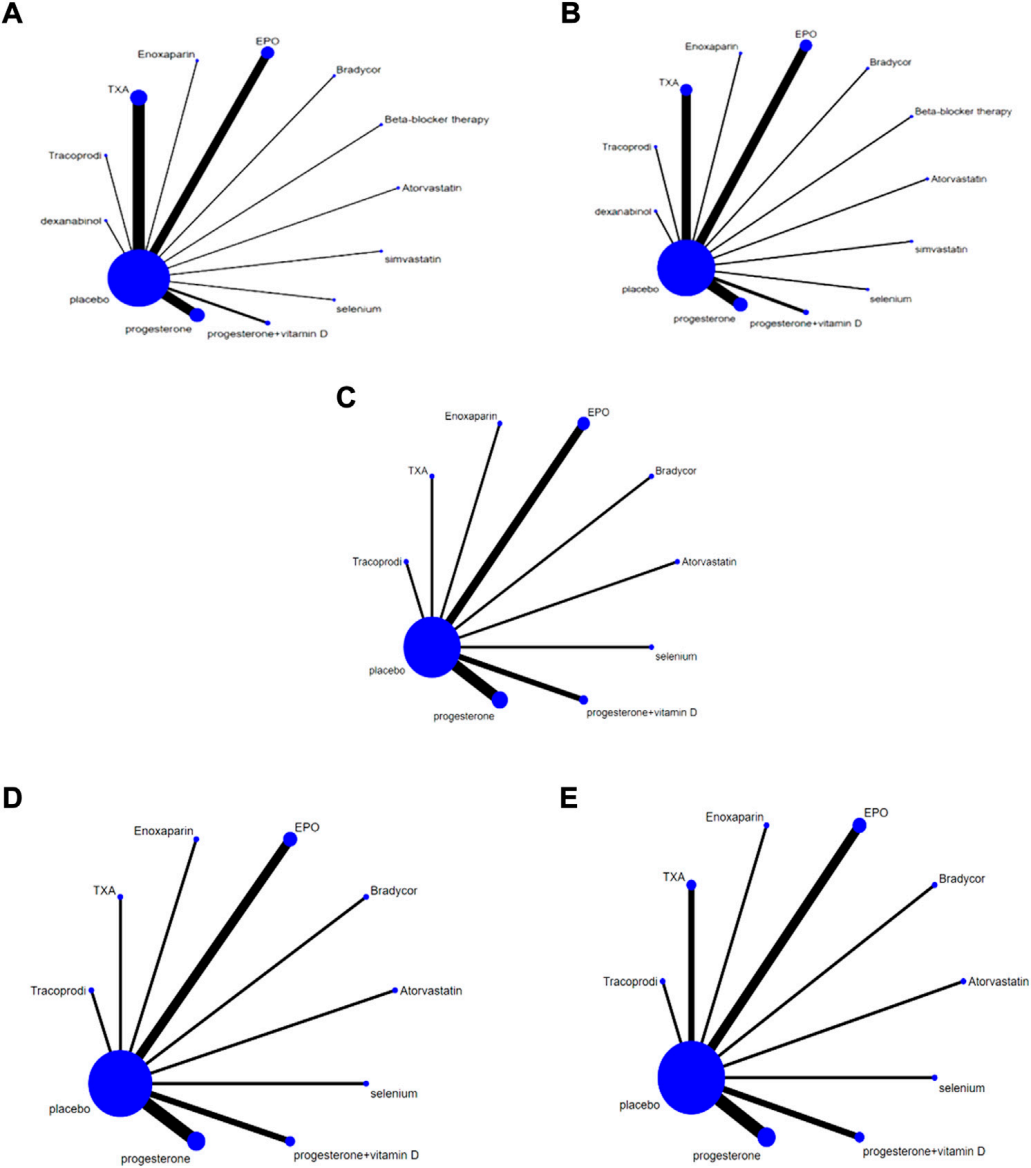
Network Chart; **(A)** Network chart based on the mortality of TBI; **(B)**. Network chart based on the patient proportion of the favorable result of TBI; **(C)**. Network chart based on the patient proportion of the good recovery of TBI; **(D)**. Network chart based on the patient proportion of the moderate disability of TBI; **(E)**. Network chart based on the patient proportion of the severe disability of TBI.

A direct line between the two intervention groups indicated evidence of direct comparison, while no line indicated the lack of direct comparison evidence. The size of dots in the figure represents the sample size, and the thickness of lines represents the number of studies. No direct comparative evidence was found in the trials included in this study. The drug treatment schemes were indirectly compared using placebo as a medium, including 13 interventions: TXA, EPO, progesterone, progevitamin + vitamin D, atorvastatin, beta-blocker therapy, Bradycor, Enoxaparin, Tracoprodi, dexanabinol, selenium, simvastatin, and placebo (Figures 4A–E).

#### 3.4.2 Sequence diagram of network meta-analysis

**FIGURE 5:**
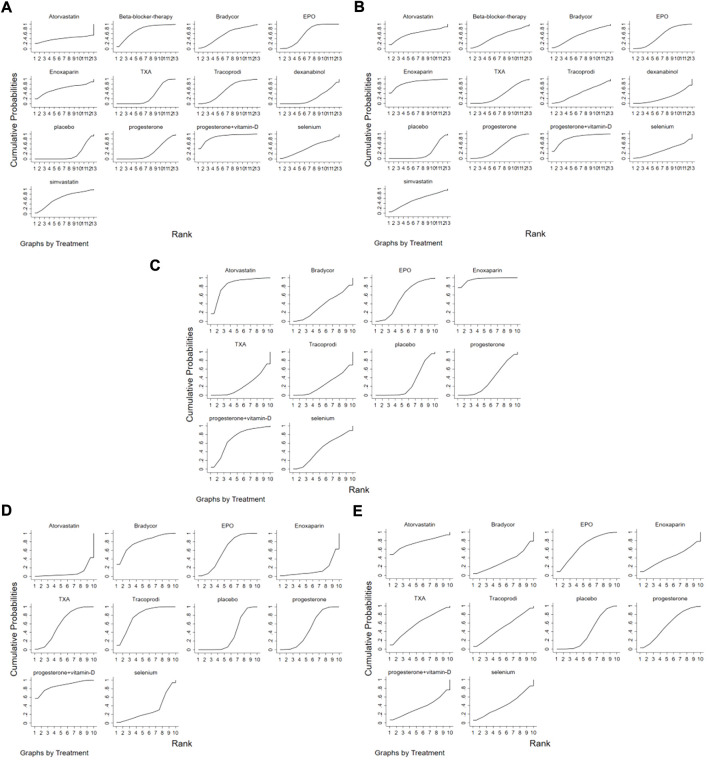
The Rank Chart; **(A)** The rank chart based on the mortality of TBI; **(B)**. The rank chart based on the patient proportion of the favorable result of TBI; **(C)**. The rank chart based on the patient proportion of the good recovery of TBI; **(D)**. The rank chart based on the patient proportion of the moderate disability of TBI; **(E)**. The rank chart based on the patient proportion of the severe disability of TBI.

The analysis of the mortality of patients with TBI after different drug treatments, involving 13 different drug treatment regimens (Figure 5A). In this figure the larger area under the curve, this drug treatment regimen has a lower mortality, it revealed that each drug treatment intervention significantly reduced the mortality of patients with TBI compared with placebo treatment. The mortality rates were ranked from the lowest to the highest: progesterone + vitamin D, beta-blocker therapy, EPO, simvastatin, Enoxaparin, Bradycor, Tracoprodi, selenium, atorvastatin, TXA, progesterone, dexanabinol, and placebo. Further, the analysis in terms of the proportion of patients with TBI who achieved favorable outcomes (Figure 5B). In this figure, the larger area under the curve means that this drug treatment regimen has a better favorable outcome. It revealed that in patients with TBI, each drug treatment intervention significantly improved the prognosis of patients compared with placebo; the order from the highest to the lowest was Enoxaparin, progesterone + vitamin D, atorvastatin, simvastatin, Bradycor, EPO, beta-blocker therapy, progesterone, Tracoprodi, TXA, selenium, dexanabinol, and placebo.

Subsequently, based on the classification of GOS score levels after each drug treatment, this research group performed analysis from three aspects: good recovery, moderate disability, and severe disability, involving 10 treatment schemes. The Figure 5C shows the network meta-analysis sequence diagram for the patient proportion of the good recovery of TBI. In this figure the larger area under the curve, this drug treatment regimen has a higher proportion of the good recovery. In terms of good recovery revealed that the proportion of patients recovering well after treatment with Enoxaparin, atorvastatin, progesterone + vitamin D, EPO, selenium, Bradycor, and progesterone increased compared with placebo. However, Tracoprodi and TXA regimens had a lower proportion of patients recovering well compared with placebo regimens; the order from the highest to the lowest was Enoxaparin, atorvastatin, progesterone + vitamin D, EPO, selenium, Bradycor, progesterone, placebo, Tracoprodi, and TXA. The Figure 5D shows the network meta-analysis sequence diagram for the patient proportion of the moderate disability of TBI. In this figure the larger area under the curve, this drug treatment regimen has a higher proportion of the moderate disability. In terms of moderate disability revealed that progesterone + vitamin D, Bradycor, Tracoprodi, EPO, TXA, and progesterone regimens had a higher proportion of patients with moderate disability compared with placebo. However, selenium, Enoxaparin, and atorvastatin had a lower proportion of patients with moderate disability compared with placebo; the order from the highest to the lowest was progesterone + vitamin D, Bradycor, Tracoprodi, EPO, TXA, progesterone, placebo, selenium, Enoxaparin, and atorvastatin. The Figure 5E shows the network meta-analysis sequence diagram for the patient proportion of the severe disability of TBI. In this figure the larger area under the curve, this drug treatment regimen has a higher proportion of the severe disability. This figure revealed that atorvastatin, EPO, TXA, progesterone, Tracoprodi, Enoxaparin, and selenium had a higher proportion of patients with severe disability compared with placebo. However, the progesterone + vitamin D and Bradycor regimens had a lower proportion of patients with severe disability compared with placebo; the sequence from high to low was atorvastatin, EPO, TXA, progesterone, Tracoprodi, Enoxaparin, selenium, placebo, progesterone + vitamin D, and Bradycor.

## 4 Discussion

The lasting health effects of TBI can lead to damaging functional limitations and reduced quality of life, which may include cognitive decline as well as obstacles involving memory, attention, behavior, and emotion (Sulhan et al., 2020). TBI provides evidence of changes in brain function or brain pathology, mainly caused by car accidents, falls, and assaults. The severity of the resulting injury depends on many factors, including the nature of the initiating force, impact location, and magnitude (Flanagan, 2015; Zhou-Guang et al., 2015). However, the clinical outcome of TBI is not limited to the original event. In the following hours to days after TBI, the immune cells release a variety of signal molecules and inflammatory factors following neuronal and glial dysfunction, metabolic changes, nerve inflammation, and cerebral edema, leading to blood–brain barrier damage, cerebral hypoperfusion, mitochondrial dysfunction, and oxidative injury. This is known as secondary injury (Winkler et al., 2016; Dorsett et al., 2017; Simon et al., 2017; Sorby-Adams et al., 2017). With the continuous understanding of the pathophysiological evolution after TBI, the researchers discovered that secondary injury might be more destructive than primary injury. Therefore, finding effective treatment options for secondary injury may be a potential therapeutic target to improve the prognosis of such patients.

In recent years, neuroscientists have explored a number of neuroprotective medicines, but they have little effect. At present, a wide variety of neuroprotective drugs are used after TBI, but the evaluation and recognition of the therapeutic effects of these drugs are uneven. Also, which drugs are more suitable for these patients is unclear. This research group used the principle of indirect comparison of network meta-analysis to comprehensively search existing RCTs on drug therapy after TBI. Finally, 30 RCTs were included for analysis, including a total sample size of 26,956 cases and 13 kinds of intervention measures. The analysis revealed that each of these drugs significantly reduced mortality in patients with TBI and increased the proportion of patients with favorable outcomes after TBI compared with placebo. Subsequently, based on the classification of GOS after each drug treatment, this study also analyzed three aspects: good recovery, moderate disability, and severe disability, involving 10 treatment regimens. It found that progesterone + vitamin D, Bradycor, EPO, and progesterone all had a higher proportion of patients with good recovery and moderate disability compared with placebo, but a lower proportion of patients with a severe disability after progesterone + vitamin D and Bradycor treatment.

Among the interventions, progesterone + vitamin D, beta-blocker therapy, simvastatin, enoxaparin, Bradycor, Tracoprodi, selenium, atorvastatin and dexanabinol were used in only one RCT each. And the sample size of these was also very small (20, 99, 49, 26, 66, 198, 57, 21, and 428, respectively). Based on the aforementioned results, we speculated that progesterone + vitamin D treatment might be the most suitable treatment strategy for patients with TBI. However, we still believe that the result was unreliable due to the small sample size, and hence needs to be validated using a large number of high-quality RCTs.

This study examined the remaining RCTs for the effectiveness of TXA, EPO and progesterone in patients with TBI. The TXA was used in 8 RCTs, with a sample size of 10674. Our study showed that TXA reduced the mortality in these patients and improved their favorable outcomes. However, Lawati et al. (2021), showed an inconsistent result; they revealed that TXA probably had no effect on mortality or disability in these patients. The reason for this inconsistency that their study focused on ICU-related brain injury. Also, the study by CRASH-2 collaborators exploring the effectiveness of TXA in patients with moderate-severe TBI was not included. The intervention using progesterone was applied in 7 RCTs and with a sample size of 1,296. Our result showed that progesterone was reduced the mortality in these patients and improved their favorable outcomes. The result was similar to the findings of Pan et al. (2019) and Zhang et al. (2020), who indicated that progesterone administration was associated with a lower mortality and improved the clinical outcomes. Additionally, Lu et al. (2016), also revealed that progesterone improved neurologic outcome in these patients but did not decrease mortality. Therefore, the effect of this drug on mortality also needed further exploration. The last 6 RCTs explored the effectiveness of EPO in such patients, and the sample size was 789. Our result showed that EPO also reduced the mortality in these patients and improved their favorable outcomes. This was similar to the findings of Lee et al. (2019) and Liu et al. (2017), who showed that EPO might prevent the death of these patients. However, the improvement in neurological outcome(s) did not reach statistical significance. Hence, this effect of EPO remains unclear and needs further investigation.

## 5 Limitations

Several limitations of this network meta-analysis need to be considered to better explain the results of this study and make this analysis provide some reference value in clinical treatment. First, only the EPO, TXA, and progesterone groups had a large sample size, and large-scale high-quality randomized controlled trials were included in the analysis. However, the remaining nine interventions involved only one RCT with a small sample size, and their results were unreliable. A further summary analysis is still needed after a large number of relevant large-scale high-quality RCTs are published. Second, the overall incidence of drug treatment–related adverse effects were not given for all interventions included in the analysis. Therefore, this network meta-analysis did not conduct a clustering analysis in terms of both effectiveness and safety. Whether the existence of adverse effects affected the ranking of their associated treatment effectiveness was not determined. In addition, whether blinding and allocation concealment were correctly implemented in some of the 30 RCTs included in this analysis was unclear. Moreover, blinding and allocation concealment were not implemented in two trials, which also reduced the strength of evidence in this analysis to some extent. Therefore, further exploration is still needed to remedy these deficiencies so as to draw more precise and convincing conclusions to guide clinical treatment.

## 6 Conclusion

The analysis of this study revealed that in patients with TBI, TXA, EPO, progesterone, progesterone + vitamin D, atorvastatin, beta-blocker therapy, Bradycor, Enoxaparin, Tracoprodi, dexanabinol, selenium, and simvastatin reduced mortality and increased the proportion of patients with favorable outcomes compared with placebo. The progesterone + vitamin D regimen had not only a high proportion of patients with good recovery and moderate disability but also a low proportion of patients with severe disability and mortality. However, the feasibility of this intervention for clinical promotion needs further exploration.

## Data Availability

The original contributions presented in the study are included in the article/Supplementary Material, further inquiries can be directed to the corresponding authors.
