# Supplementary material for: Effect of drug therapy on nerve repair of moderate-severe traumatic brain injury: A network meta-analysis
Source: Front Pharmacol. 2022 Nov 2;13:1021653. doi: 10.3389/fphar.2022.1021653 (PMC9666493; doi:10.3389/fphar.2022.1021653)
Supplement: Supplementary file 1 [file DataSheet1.docx]

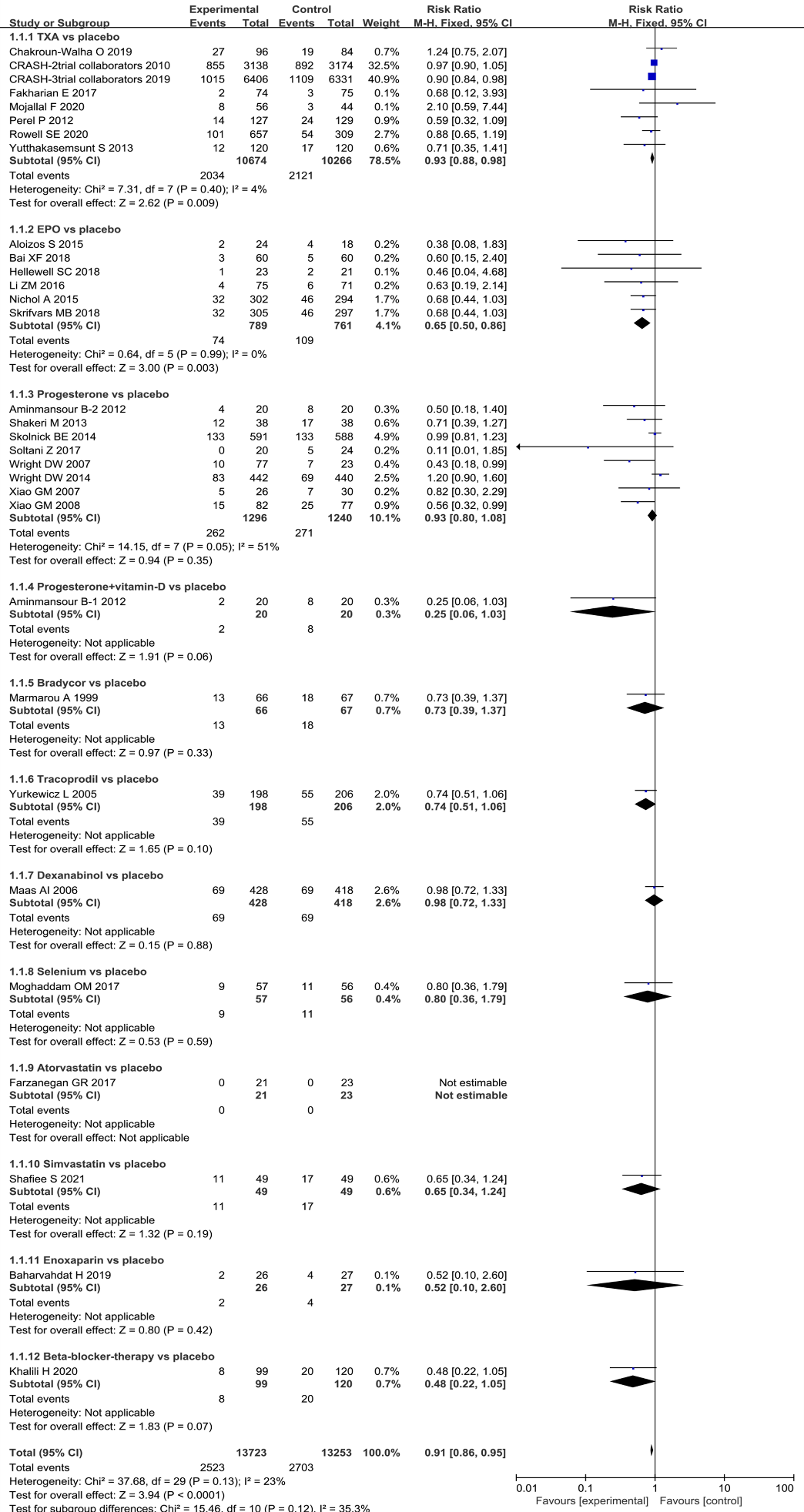


Supplementary Figure 1a. Subgroup analysis of the mortality of TBI


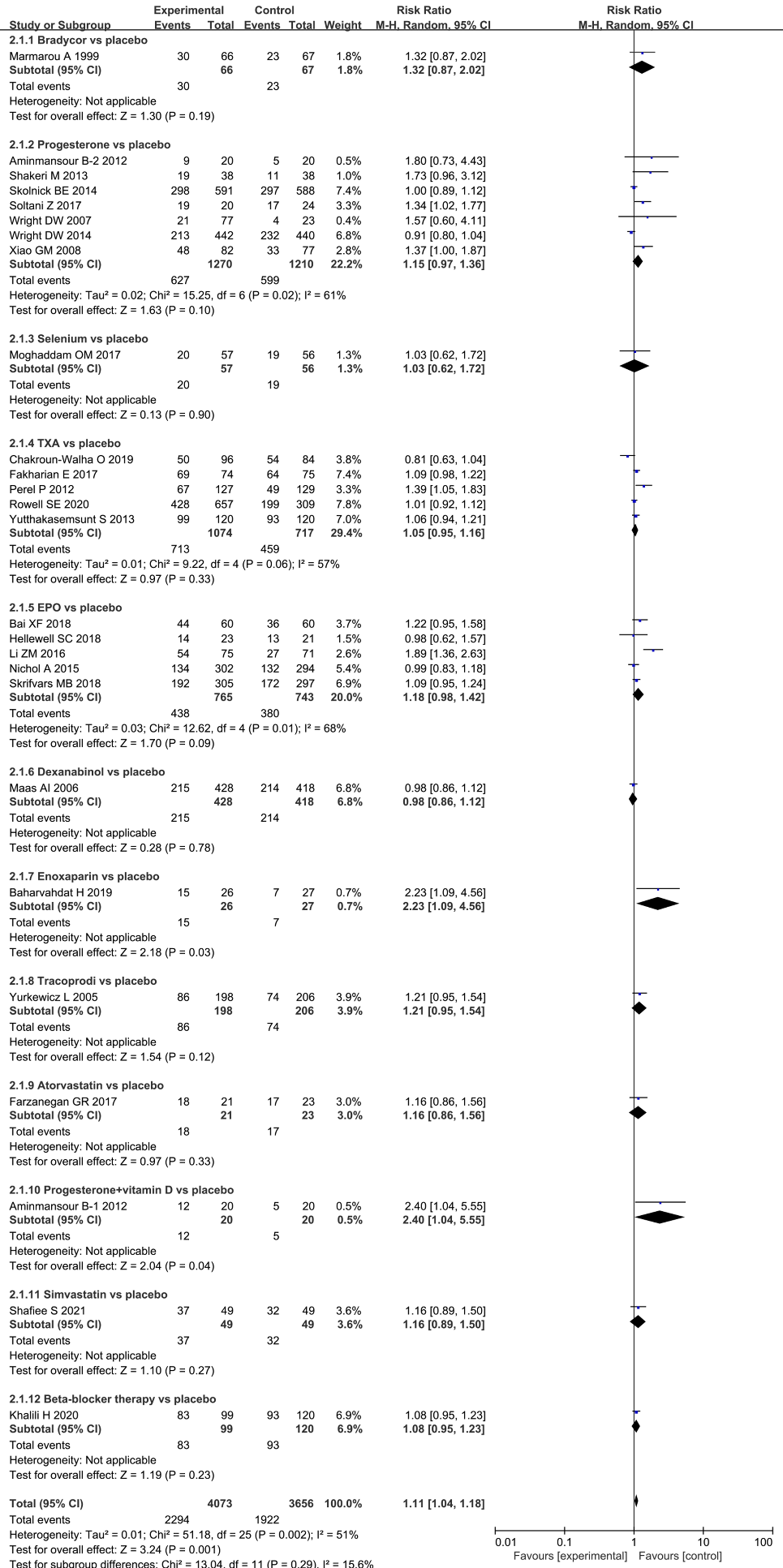


Supplementary Figure 1b. Subgroup analysis of patient proportion of the favorable result of TBI
